# Formation Mechanism and Dynamic Evolution Laws About Unsafe Behavior of New Generation of Construction Workers Based on China’s Construction Industry: Application of Grounded Theory and System Dynamics

**DOI:** 10.3389/fpsyg.2022.888060

**Published:** 2022-04-26

**Authors:** Guodong Ni, Lei Lv, Shaobo Wang, Xinyue Miao, Yaqi Fang, Qing Liu

**Affiliations:** ^1^School of Mechanics and Civil Engineering, Research Center for Digitalized Construction and Knowledge Engineering, China University of Mining and Technology, Xuzhou, China; ^2^School of Mechanics and Civil Engineering, China University of Mining and Technology, Xuzhou, China; ^3^School of Civil Engineering, Xuzhou University of Technology, Xuzhou, China

**Keywords:** unsafe behavior, formation mechanism, dynamic evolution laws, new generation of construction workers, behavior motivation, grounded theory, system dynamics

## Abstract

Construction workers’ unsafe behavior is a major cause of safety accidents and injuries, therefore, a profound understanding of the formation process and evolution laws about construction workers’ unsafe behavior is conducive to taking measures to prevent incidents. At present, the new generation of construction workers (NGCWs) born after 1980 are gradually becoming the main force at construction sites in China. Given that generational differences of construction workers can cause the discrepancies in their thoughts and attitudes when engaging in safety-related activities, this study aims to investigate the formation mechanism and dynamic evolution laws about NGCWs’ unsafe behavior based on the context of China’s construction industry. From the perspective of behavior motivation, in-depth semi-structured interviews with 18 NGCWs and 7 grassroots managers were conducted, and data analysis followed a three-step coding process based on grounded theory. Through continuous comparison, abstraction and analysis, the stimulus-organism-response theory was introduced and expanded to construct a three-stage formation mechanism model. On this basis, the causal diagram and stock flow diagram were developed based on system dynamics principles to reflect the dynamic feedback relationships of the factors in the static formation mechanism model, and simulation was carried out using Vensim PLE software. The results show that three types of internal needs and three types of external incentives stimulate corresponding motivations for NGCWs’ unsafe behavior. Two types of individual factors, five types of situational factors and behavior result play an influencing role in the decision-making process of externalizing motivation into behavior. Under the synergistic effect of multiple factors, the level of unsafe behavior displays a downward trend, and the rate of decrease is slow first and then fast. Furthermore, among individual factors and situational factors, safety awareness and safety management system have the most significant effect on the level of unsafe behavior, while situational factors play a more obvious role. The findings can provide theoretical support and practical references to China’s construction companies and government departments for the purpose of improving NGCWs’ unsafe behavior.

## Introduction

Due to high accident and casualty rates, the construction industry has long been regarded as one of the most dangerous industries (Im et al., 2009; Abbas et al., 2018; Hasanzadeh et al., 2019). China’s construction industry is likewise in a precarious state in terms of safety. From 2010 to 2019, around 7275 construction workers died in workplace accidents, with an average of 1.99 deaths per day (Xu and Xu, 2021). Furthermore, since 2015, the number of safety accidents and fatalities in housing and municipal construction has shown an increasing trend year by year (Zhang J. et al., 2020). The occurrence of accidents can have a detrimental influence on workers, their families, the organizations and even the whole society (Peng et al., 2015). It is clear that the safety production situation in this sector in China is still severe and complicated, and the level of safety management needs to be improved further. Unsafe behavior is a major cause of injuries at construction sites, which leads to approximately 80%–90% of all accidents (Han and Lee, 2013). Construction workers, as the ultimate executors of construction projects and the primary victims of accident injuries, are fundamental to behavioral safety and the most critical aspect in safety management (Liu et al., 2020). Therefore, reducing the prevalence of construction workers’ unsafe behavior remains an effective way to carry out incident prevention.

As a typical labor-intensive industry, the construction industry provides employment for about 220 million people worldwide, including a substantial share of migrant workers (Shepherd et al., 2021). This is especially noticeable in China. Driven by the wide urban-rural income disparity and attracted by the low requirements of job seekers’ educational background and professional skills, large numbers of migrant workers have treated the construction industry as a common destination industry (Jiang et al., 2020). According to the monitoring survey report on migrant workers in 2020 released by National Bureau of Statistics of China, the construction industry accounts for 18.3% of the employment of migrant workers, second only to the manufacturing industry; in terms of age, migrant workers aged 40 and below account for 49.4% of all (National Bureau of Statistics of China, 2021). Because of *hukou* (household registration) system, more than 90% of construction workers are migrant workers (Swider, 2015). As a result, as current demand for construction labor force increases (Man et al., 2021) and the old construction workers return to their hometowns for retirement, the construction workers born after 1980 are gradually becoming the main force at construction sites (Ni et al., 2020). Previous research has revealed that workers who are younger and have less job experience are more prone to engage in unsafe behavior (Qiao et al., 2018). Younger workers, in particular, have higher occupational injury rates (Salminen, 2004; Breslin and Smith, 2005; Sámano-Ríos et al., 2019), and thus are in greater need of occupational safety and health services (Dragano et al., 2018). Additionally, numerous statistics and studies have indicated that migrant construction workers are more likely to be involved in safety accidents than native construction workers (e.g., Kim J. M. et al., 2020; Shepherd et al., 2021). In light of dual effect of times and industry, it is vital to pay more attention to the safety of young generation of construction workers in China.

In view of the key role of the post-80s migrant workers in the process of urbanization, the Chinese authorities first proposed the idea “new generation of migrant workers” in Document No. 1 of the Chinese Communist Party Central Committee and the State Council in 2010, and stressed that specific measures must be taken to solve the problem (Franceschini et al., 2016). In this manuscript, the new generation of construction workers (NGCWs) refer to workers and laborers who were born after 1980, aged 16 and above, and engaged in front-line construction work. They mainly consist of new generation of migrant workers who work in cities and registered in rural areas. Unlike their parents, NGCWs were born during a period of reform and opening up, and most of them are also the first one-child generation. A rising standard of living, an increasingly open environment, and fewer life adversities and setbacks have built their significant distinctions. NGCWs are usually better educated and have higher overall quality than the old generation (Lin and Mai, 2018). They are more concerned with quality of life and aspire to urban lifestyles (Franceschini et al., 2016; Zheng, 2021), therefore, the majority of them leave for cities and spend more time on non-agricultural activities (Zhao et al., 2018). Besides, they pursue freedom and have an adventurous spirit, but lack the capacity to cope with stress and are reluctant to suffer, presenting major features such as strong emotional oscillation, low job satisfaction, and resistance to rules and constraints (Cennamo and Gardner, 2008; Lin and Mai, 2018; Fang et al., 2020). Different generations are believed to share various personality characteristics in terms of values and preferences due to diverse upbringing circumstances. These differences pose significant challenges for organizational management, because groups with different values often vary in their workplace attitudes and behaviors (Dencker et al., 2008). According to survey statistics, 80% of occupational accidents are related to the post-80s in China (Gao et al., 2015). It is evident that traditional safety management measures have limited effect on reducing the occurrence of NGCWs’ unsafe behavior (Gao et al., 2016; Ni et al., 2020). Companies and organizations should modify their practices and regulations to meet generational disparities in work values and behaviors (Wey Smola and Sutton, 2002); nevertheless, adjustments to address NGCWs’ unsafe behavior are still rare in the construction industry.

To ameliorate the current situation, it is necessary to explore the causes of NGCWs’ unsafe behavior. However, previous studies seem to neglect the particularity of NGCWs. Moreover, the majority of these studies only focused on the relationships between antecedents and unsafe behavior, such as emotional exhaustion (Ju et al., 2016), safety habits (Mohajeri et al., 2021), and perceived safety climate (Seo, 2005). Although these research hypotheses contribute to the establishment of theoretical models and have some theoretical significance, there are still certain limitations. On the one hand, most of these factors are not the direct cause of unsafe acts; on the other hand, the relevant antecedents derived from the exploration are more fragmented and lacking in systematization, so inevitably have the disadvantages of singularity and one-sidedness in the face of complex construction environment. Research on formation mechanism of unsafe behavior is a deepening on the basis of influencing factors, which overcomes the shortcomings of the analysis of a single factor. The formation of unsafe behavior is a relatively complicated process, and clarifying the formation mechanism of unsafe behavior assists managers and workers in making specific preparations for controlling unsafe behavior, which is of great significance to on-site management practice (Fang et al., 2016).

Unsafe behavior that leads to accidents is often intentional (Donald and Cantor, 1993), which shows that even if workers are aware of the hazardous situation, they still choose unsafe acts (Fang et al., 2016). Behavior motivation is an essential perspective for understanding the formation mechanism of unsafe behavior. It plays a vital function in determining the intensity and direction of behavior (Quested et al., 2017), which considered a direct driver of human acts. Therefore, in many fields, motivation has been recognized as a significant element in the prediction of behavior (e.g., Mattingly et al., 2012; Ha and Ng, 2015). In general, motivation, knowledge and ability are three determinants of work behavior, however, in normal work situations, whether the work can be done safely may be determined more by motivation because employees can usually acquire the pre-requisite knowledge and skills needed via selection and training (Andriessen, 1978).

In the field of safety science, current research on motivation still focuses on the measurement and examination of the influence between two variables (e.g., Panuwatwanich et al., 2017; Lim et al., 2018; Ansori et al., 2021; Mohajeri et al., 2021). Little scholarly attention has been paid to the formation mechanism of construction workers’ unsafe acts from the perspective of motivation, that is, the pathway between motivation and unsafe behavior of construction workers has not been sufficiently portrayed. Additionally, generational differences caused by different social environments in which the workers grow up ought to be fully considered, however, the NGCWs with distinctive era characteristics have not received more research support in terms of their unsafe behavior. Moreover, it is necessary to explore the dynamic evolution laws about construction workers’ unsafe behavior in order to intuitively analyze its changes. Therefore, this study aims to specifically analyze and portray the formation mechanism of NGCWs’ unsafe behavior from the perspective of behavior motivation using grounded theory, and this process also benefits from the stimulus-organism-response (SOR) theory which can offer a suitable framework to explain how unsafe behavior occurs. Following that, the current study explores the dynamic evolution laws, developmental stages and key influencing factors of NGCWs’ unsafe behavior as well. It is expected that the findings can fill the research gap, enrich the literature on construction workers’ unsafe behavior and provide a new insight to improve safety performance at construction sites in China.

## Formation Mechanism of New Generation of Construction Workers’ Unsafe Behavior

### Research Design

#### Research Method

In this study, a grounded theory approach was utilized to explore the formation mechanism of NGCWs’ unsafe behavior. Grounded theory is a scientific qualitative research method, which mainly observes a phenomenon and aims to develop a theory based on the data systematically collected and analyzed (Urquhart et al., 2010). This method emphasizes the systematic data analysis program, and extracts the core concepts and categories of the original data via repeated comparison, analysis and refinement (Lyu, 2020). Moreover, it offers excellent support for abstracting and relating categories to each other through different coding processes (Goldkuhl and Cronholm, 2010). Most importantly, given the scarcity of research on the reason for NGCWs’ unsafe behavior, grounded theory could provide a novel methodological design to enhance the understanding of how workers’ perspectives, attitudes, and behavior are constructed in specific personal and social contexts (Narushima and Sanchez, 2014). Therefore, a three-level coding procedure (including open coding, axial coding, and selective coding) (Strauss and Corbin, 1998) was applied to analyze original data, extract key elements of the formation process and analyze the interaction mechanism, so as to make up for the defects that quantitative research is not suitable for digging deeper into the phenomenological information.

#### Participants

Considering that self-reports and personal impressions or observations of others’ behavior are all valuable sources of information (Nübold et al., 2017), the researchers will approach and collect data from NGCWs and grassroots managers who are willing to report on themselves and someone else. On the one hand, it avoids participants to conceal themselves during the interview because of touching on sensitive topics that may bring them trouble; on the other hand, grassroots managers are more familiar with the construction sites and may be more likely to reveal how NGCWs’ unsafe behavior occurs from an objective perspective. To ensure the scientific validity and high heterogeneity of data sources, this study did not restrict the workplaces and job types of the participants. Additionally, the NGCWs interviewed were limited to those born after 1980, and there was no restriction on the age of managers. Moreover, the researchers did not determine the sample size in advance, but kept collecting data. In this process, theoretical sampling strategy was employed until the theory reached saturation, i.e., no new categories and relationships emerged. Although the value of qualitative studies may depend more on the quality of the data than the size of sample (Ni et al., 2021), the number of participants in this manuscript is in line with the recommendation of 20–30 for grounded theory (Nübold et al., 2017). Ultimately, theoretical saturation was reached after conducting 25 interviews, including 18 (72%) NGCWs and 7 (28%) grassroots managers. All participants are male, and the basic information of participants is shown in Table 1.

**TABLE 1:** Basic information of participants.

Number	Position	Age (year)	Experience (year)	Educational background	Project location
A_01_	Construction crew	26	5	Undergraduate	Hubei
A_02_	Project manager	49	20	High School	Fujian
A_03_	Site supervisor	28	3	Undergraduate	Shandong
A_04_	Safety inspector	28	4	Undergraduate	Shandong
A_05_	Foreman	26	7	Secondary specialized school	Jiangsu
A_06_	Safety inspector	28	5	Undergraduate	Shandong
A_07_	Site supervisor	30	4	Undergraduate	Jiangsu
B_01_	Worker man	30	10	Junior high school	Hebei
B_02_	Electric welder	26	6	Secondary specialized school	Jiangsu
B_03_	Electric welder	32	10	Secondary specialized school	Jiangsu
B_04_	Carpenter	37	15	Junior high school	Jiangxi
B_05_	Tower crane operator	26	6	Primary school	Anhui
B_06_	Bricklayer	26	10	Junior high school	Hunan
B_07_	Scaffolder	30	10	Junior high school	Jiangsu
B_08_	Plasterer	33	10	Junior high school	Jiangxi
B_09_	Wall and floor tiler	37	14	Junior high school	Jiangsu
B_10_	Carpenter	31	10	Junior high school	Fujian
B_11_	Painter	33	8	Junior high school	Jiangsu
B_12_	Reinforcing bar worker	35	12	Junior high school	Jiangxi
B_13_	Plasterer	28	5	Junior high school	Jiangxi
B_14_	Scaffolder	29	7	Junior high school	Shandong
B_15_	Tower crane operator	26	8	Secondary specialized school	Jiangsu
B_16_	Painter	38	18	Primary school	Henan
B_17_	Carpenter	34	10	Junior high school	Guangdong
B_18_	Painter	29	7	Junior high school	Henan

*A_i_ represents grassroots managers; B_j_ represents NGCWs.*

#### Data Collection

In-depth semi-structured interviews were conducted with participants to collect the data needed. The in-depth qualitative interview is particularly suitable for grounded theory because both of them are open and oriented (Charmaz, 2006). Meanwhile, the semi-structured interview has the advantage of being two-way interactive, and it allows interviewers to flexibly adapt and add additional questions based on the answers given by the interviewees to explain in more depth how the person experienced. In particular, prompts can be given when interviewees fail to answer or deviate from the topic to ensure that the conversation can continue, which avoids the problem that individual literacy or understanding bias may affect the judgment of the questions in traditional questionnaires.

The data collection was conducted mainly in the form of phone calls and WeChat, supplemented by on-site interviews. The interview team comprised of 2–3 researchers, with one leading the interview process, one recording the relevant information in real time using a tape recorder, and another one acting as a mobile person to participate in the interview process when needed. In order to improve efficiency, the whole process was conducted around outlines which help to guide participants to fully express their opinions and viewpoints on the subjects. The questions in the outlines are based on relevant literature (e.g., Man et al., 2017), and specific outlines are presented in Appendices A, B. Each interview lasted approximately 50–70 min. After an interview, the collected audio data was converted into initial text data in a timely manner.

### Data Analysis

#### Open Coding

Open coding is the process of abstracting different concepts from the original utterance data and merging concepts with similar meanings into subcategories (Lyu, 2020). The researchers imported interview data into NVivo 12.0 software for coding and strictly adhered to the coding procedure. During the coding process, a line-by-line and sentence-by-sentence reading was taken to mark the information closely related to the purpose of this study, and to distill and summarize it into concepts. Following that, the connections between concepts were further explored, and concepts with interrelated meanings were grouped into subcategories. In order to avoid subjective bias, the subcategories were named by extracting the original words of the participants, but also by drawing on relevant literature for summarizing and refining. In addition, constant comparison and revision were required to identify similarities and variances among participants. In this work, 169 concepts and 28 subcategories were formed by repeated comparison, integration, and generalization. Table 2 shows an excerpted sample of refinement and induction process of open coding, and 2–3 concepts with the highest frequency of occurrence were selected from each subcategory for display.

**TABLE 2:** Subcategories and concepts developed from original interview data through open coding (excerpted sample).

Number	Subcategory	Concept	Original interview data
1	Pursuit of comfort	Laziness	A_03_ “It is common for lazy workers to fail to take the safety precautions before work.”
		Discomfort	B_03_ “You know it is very hot in the summer. It’s uncomfortable to wear a helmet, so sometimes I don’t wear one.”
2	Weak risk perception	Underestimation of accident rates	B_01_ “You think the likelihood of an accident without a helmet is so small that you think it’s okay, is that right?” “Yes.”
		Danger perception	B_01_ “Older workers are more experienced and have a better ability to perceive danger than we do.”
		Unawareness of risks	A_05_ “First of all, the management is not in place. Second, the workers themselves are not aware of the potential risks, and the incidents at construction sites cannot be completely prevented. There is no way to deal with everything. The key is to be careful yourself.”

#### Axial Coding

The object of axial coding is to analyze the correlation between subcategories and further discover the categories (Zhao et al., 2020). Next, several dimensions or directions of the theory can be extracted. The researchers further analyzed the relationships of the 28 subcategories acquired by open coding, and finally obtained 16 categories. Some of the categories, subcategories as well as the connotation of subcategory are shown in Table 3.

**TABLE 3:** Categories developed through axial coding and the connotation of subcategory (excerpted sample).

Category	Subcategory	The connotation of subcategory
Physiological needs	Time and effort saving	Failure to perform necessary safety operations in order to save time and increase efficiency.
	Pursuit of comfort	Failure to perform necessary safety operations in order to purse comfort.
Psychological needs	Self-esteem needs	Workers’ enjoyment of performing unsafe acts to project themselves in groups; the rebellion in the face of criticism and the refusal to obey instructions due to the priority of saving face.
	Sensation seeking	A willingness to challenge oneself, the thought of risk-taking and a tendency to try unsafe acts.

#### Selective Coding

Selective coding is a process that revisits the source material after open coding and axial coding to unearth the core category and develops the integration of theoretical constructs (Draucker et al., 2007). The core category must be overarching and maximize the ability to encompass the findings within a broad theoretical scope (Zhang W. et al., 2020). That is, the goal of selective coding is to develop a single storyline around which everything else revolves (Zhou et al., 2015). And the conceptualization of relational form between categories makes the analytical story coherent and theorized (Charmaz, 2006). Through repeated investigation and analysis of the relationships between the categories, the researchers eventually re-clustered the 16 categories into five sub-core categories: internal needs, external incentives, situational factors, individual factors and behavior result. This storyline also emerged: NGCWs’ motivations for unsafe behavior, which are stimulated by internal needs and external incentives, are the direct cause of their unsafe behavior; individual and situational factors regulate the link between motivation and behavior, and thus influence the decision-making process of externalizing motivation into behavior; unsafe behavior is closely related to safety accidents which considered a potential result, and whether or not an incident occurs may further influence the decision-making process. Based on this storyline, the core category “the formation mechanism of NGCWs’ unsafe behavior” was proposed. The coding results are shown in Table 4.

**TABLE 4:** Coding results.

Core category	Sub-core category	Category	Subcategory	Concept
Formation mechanism of NGCWs’ unsafe behavior	Internal needs	Physiological needs	Time and effort saving	Thought of finishing work early, pursuit of efficiency, etc.
			Pursuit of comfort	Laziness, discomfort, etc.
		Psychological needs	Self-esteem needs	Pursuit of a “tough guy” image, concern for self-esteem, bravado, etc.
			Sensation seeking	Frequent risky attempts, curiosity, etc.
		Economic needs	Increase of income	Thought of making more money, thought of working more to earn more, etc.
	External incentives	Work stress	Schedule pressure	Hurry at work, schedule compression, deadline, etc.
			Leadership pressure	Leadership arrangements, fear of leadership displeasure, etc.
		Group norms	Expedient conformity	Silence of co-workers on unsafe behavior, silence of managers on unsafe behavior, etc.
			Blind conformity	Simple imitation of older workers, conformity, etc.
		Poor quality of social exchange relationships	Discord with workmates	Frequent disputes, fights, etc.
			Destructive leadership	Accusation in public, failure to deliver on promises, indifference on workers, etc.
			Weak sense of belonging	Weak relationship connection, high mobility, etc.
	Situational factors	Safety climate	Safety management commitment	Failure to lead by example, leadership non-compliance with safety regulations, profit orientation, etc.
			Workers’ safety participation	Little safety communication, non-reporting of accidents, etc.
		Safety management system	Safety regulations	Inappropriate safety procedures, improper work practices, etc.
			Safety supervision	Insufficient safety inspection, failure to impose penalties, etc.
			Safety training	Explanation of safety knowledge, training of safety skills etc.
	Individual factors	Unsafe psychology	Fluke psychology	Luck, fluke, etc.
			Paralysis psychology	Empiricism, paralysis, etc.
		Safety risk perception	Weak risk perception	Underestimation of accident rates, danger perception, unawareness of risks, etc.
		Self-efficacy	High self-confidence	Thought of few hazardous situations, frequent risky attempts, etc.
		Safety awareness	Weak safety awareness	Habitual failure to wear safety equipment, low awareness of precautions, lack of awareness of the importance of safety, etc.
		Work experience	Little work experience	Lack of familiarity with the work, little experience of accidents, etc.
	Behavior decision-making		Choice of safety behavior	Choice of observation of safety rules and regulations, choice of wearing protective equipment, etc.
			Choice of unsafe behavior	Choice of not wearing a dust mask, choice of sitting on the protective railing to rest, etc.
	Behavior result	Safety accident	Accident	Occurrence of accidents, absence of accidents, lessons from accidents, etc.
	Behavior result feedback		Positive feedback	Tendency to violate regulations next time, tendency to perform unsafe acts next time, etc.
			Negative feedback	Lessons learned, tendency to wear a helmet next time, etc.

### Construction and Explanation of Formation Mechanism Model

#### Construction of Formation Mechanism Model

**FIGURE 1:**
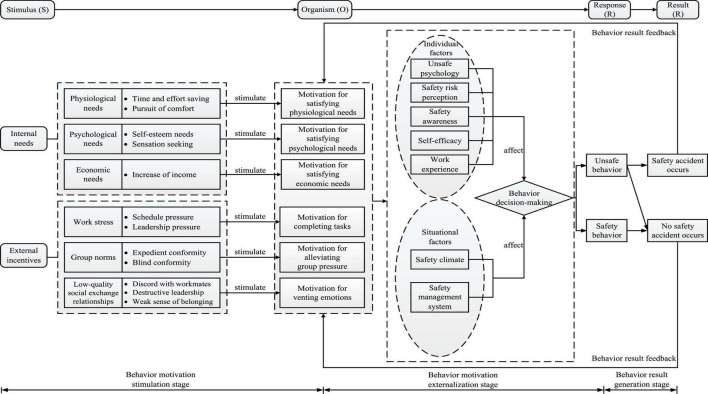
Formation mechanism model of new generation of construction workers (NGCWs’) unsafe behavior.

The SOR model is derived from the field of environmental psychology, and it is usually used to describe the relationship of the stimulus (S) received by the individual, the internal evaluation of the organism (O) and the response (R) produced by the individual (Mehrabian and Russell, 1974). “Stimulus” mainly refers to the surroundings that an individual encounters at a specific time (Jacoby, 2002), which may include the external environment as well as the physical and psychological internal environment (Lai, 2010). “Organism” refers to the emotional and cognitive intermediary states that occur when an individual interacts with stimuli (Tang et al., 2019). According to this model, environmental factors can stimulate human emotion and cognition (Kim M. J. et al., 2020). It shows that the stimulus reinforces an individual’s internal state (Eroglu et al., 2001). Finally, the individual makes behavior responses, that is, behaves in an approach or avoidance manner (Floh and Madlberger, 2013), because the reinforcements are positive or negative. The SOR model takes both objective environmental and subjective psychological factors into consideration, and it may reflect psychological states and behavior changes of individuals in response to stimuli, which is a suitable explanation for the generation of individual behavior. Therefore, this model has been widely applied to understand consumer behavior (e.g., Jacoby, 2002; Floh and Madlberger, 2013), tourism behavior (e.g., Jiang, 2022) and energy saving behavior (e.g., Tang et al., 2019). However, it has received little attention in the field of behavioral safety. The SOR theory is applicable in the present study for two reasons. First, its interpretation and understanding of complex behavior in various situations has been successfully tested by many previous studies. Second, it provides a structured theoretical framework. Based on this, the impact of the internal and external stimuli encountered by NGCWs on internal psychological state and subsequent behavior choices can be more reasonably explained. Therefore, it can offer a structured research framework as well as a solid theoretical foundation for investigating the formation process of NGCWs’ unsafe behavior. On the basis of the coding result and the storyline, the internal needs and external incentives that stimulate corresponding motivations for the organism’s (O) unsafe behavior are considered stimuli (S), and then response (R), i.e., unsafe behavior, happens. Given that the result of unsafe behavior, i.e., whether safety accident occurs, can affect the decision-making process, this manuscript introduced result (R) link to expand the SOR theory and constructed the SORR model, which fully reflects the internal logical structure of internal needs and external incentives, motivation for unsafe behavior, unsafe behavior, and behavior result. As a result, through the correspondence and integration of the expanded SORR framework and the story line, a three-stage formation mechanism model of NGCWs’ unsafe behavior was eventually constructed, which is illustrated in Figure 1.

#### Explanation of Formation Mechanism Model

##### Behavior Motivation Externalization Stage

(1) Internal needs:

The internal need is one of the main factors that stimulate NGCWs’ motivation for unsafe behavior. Many of the participants talked about unsafe behavior that they had personally experienced or heard about from the perspective of need. Saving time and effort as well as pursuit of comfort, are two prime triggers for the motivation for satisfying psychological needs. Heavy construction activities and long hours of work cause NGCWs to engage in behavior that improve efficiency and comfort, such as working at heights without safety harnesses, using human ladders to overload building materials, crossing safety guardrails, etc. The finding is consistent with Man et al. (2017), who found that saving time is the most attractive motivation for construction workers to adopt risk-taking behavior, moreover, saving energy, convenience and comfort are associated with utilitarian outcomes as well. A qualitative study by Wong et al. (2020) also confirmed that construction workers would not wear personal protective equipment (PPE) if they valued and prioritized utilitarian outcomes. Aside from physiological needs, NGCWs have a plethora of psychological needs, such as self-esteem and sensation seeking. Some grassroots managers interviewed said that NGCWs tend to perform unsafe operations because of the priority of saving face. A_03_, for example, expressed his view, “young workers want to prove that they are ‘tough guys’ and they are not fear of getting hurt.” A_06_ stated that, “even if you warn him that it is dangerous, he would not obey you since he may feel humiliated to admit that he was wrong.” One possible explanation is that NGCWs are more eager to be recognized and respected (Giddy and Webb, 2018), besides, Choudhry and Fang (2008) also found that the need to present oneself as a “tough guy” is one of the main reasons for construction workers’ unsafe behavior. Furthermore, among individual characteristics, sensation seeking refers to the need for diverse, intense and novel sensations and experiences (Zuckerman, 1979). It is directly tied to risk propensity, and people with high sensation seeking are more inclined to take risks and may suffer more harm (Hasanzadeh et al., 2020). According to the interview data, A_03_ stated that, “Some young workers work at heights without safety ropes and often sit on fall protection fences because they find it exciting.” One possible reason is that the younger generation scores higher in sensation seeking, and sensation seeking decreases with aging (Butkoviæ and Bratko, 2003). In addition, NGCWs who are in their middle and young adulthood are under pressure to start a family, buy a car and a house, and educate their children. The economic need drives them to do hazardous but highly rewarding acts, and even work with illness and fatigue in exchange for higher wage payments. For example, B_06_ said, “We are definitely here to make money, and it doesn’t matter if we are safe or not, so we work as fast as we can.” B_10_ said, “We are paid by the quantity, and we will get more money if we work fast. After all, if you don’t work, you don’t get paid.”

(2) External incentives:

The external incentive is the other cause of NGCWs’ motivation for unsafe behavior. Pressure from the leadership and the time limit for a project make NGCWs passively complete their work tasks. Previous research has revealed the negative effects of stressful schedules and leadership pressure on safety behavior (e.g., Lipscomb et al., 2008). The interview data supports this point, for instance, B_04_ said, “Although I am very tired and want to lie down for rest after a long day work, the foreman asks me to work overtime.” When asked, “Don’t you think of the possibility of danger when you are not wearing safety ropes?” B_01_ said, “Yes, I know it’s dangerous to fail to wear a safety rope. But I’m in a hurry, I need to catch up.” Group norms are a form of submission to the herd mentality, which refers to the tendency of NGCWs to abandon the principle of compliance with safety rules and regulations under the pressure of organizational environment. In this study, group norms include both in terms of expedient conformity and blind conformity, which are manifested as compromise and passive unsafe behavior. It can be found that the motivation for alleviating group pressure often leads to the malignant infection and spread of unsafe behavior, which seriously threatens the safety production at construction sites. Several participants supported this view, for example, B_04_ said, “Everyone does this (throwing cigarette butts at no-fire zones). I’m sure nothing is wrong if everyone else does.” Moreover, discord with workmates and destructive leadership make NGCWs difficult to feel support from workers and leaders. Once there is a lack of emotional connection, NGCWs are more likely to leave, and companies with high staff turnover are more prone to encounter safety problems (Jiang et al., 2015). At the same time, the high level of mobility makes it more challenging for NGCWs to develop a sense of belonging to the team and trust in their leaders and fellow workers. Over time, they may accumulate unpleasant feelings and tend to engage in violent acts to vent their emotions, which can easily lead to unsafe behavior and eventually cause serious issues (Adinyira et al., 2020).

##### Behavior Motivation Externalization Stage

Behavior motivation externalization stage is a decision-making process that is impacted by different elements. Based on the interview transcripts, five categories of individual factors (i.e., work experience, unsafe psychology, safety awareness, safety risk perception and self-efficacy) and two categories of situational factors (i.e., safety climate and safety management system) were uncovered.

(1) Work experience:

New generation of construction workers tend to be inexperienced and are not aware of the potential risks and hazards posed by unsafe behavior. Workers interviewed indicated that their assessment of behavior largely depended on previous work experience, and they would regard the option for unsafe acts as safe if they had not suffered negative repercussions. According to a study by Man et al. (2017), younger employees are more likely to engage in risky activities. The interview transcripts are also in accordance with the findings of Choudhry and Fang (2008) that less experienced people have a more shallow understanding of safety standards. Furthermore, grassroots managers believe that owing to a lack of work experience, NGCWs have insufficient ability to detect potential risks and to deal with crisis events. It seems difficult for NGCWs to carry out proper self-help, which may result in more severe injuries. The interview data also revealed that as workers’ age and length of service rise, so would their experience with safety concerns, capacity to master safety-related regulations as well as protection abilities.

(2) Unsafe psychology:

Unsafe psychology includes fluke psychology and paralysis psychology in the present study. Fluke is a gambling mentality, which is one of the main psychological causes of violations (Fu et al., 2020). One worker interviewed said that in many cases, operations against regulations happened because NGCWs believed that the occurrence of hazards was a small probability event, and they usually assumed that these operations would not be found and punished by regulators. Moreover, one manager interviewed provided his view that paralysis psychology usually caused workers to overestimate their ability and temporary experience, which made them easy to become slack, manifesting in sloppy work, non-compliance with appropriate safety regulations and a lack of concern for the quality of work. It is clear that a lack of attention and vigilance to accidents can easily lead to paralysis (Fu et al., 2020).

(3) Safety risk perception:

Safety risk perception means further judgment or consideration of the possibility and severity of safety accident consequences (Bohm and Harris, 2010). Underestimation of safety risks is common in construction workplaces (Choudhry and Fang, 2008). In general, perceived risk vary from person to person (Shin et al., 2014). The relationship between age and risk perception has received a lot of attention (e.g., Basha and Maiti, 2013; Han et al., 2019), but specific trends have not been provided. In this study, safety risk perception substantially influenced the decision-making progress, i.e., whether NGCWs engaged in unsafe acts. The majority of NGCWs are aware of the risks involved in their work; however, the risks tend to be underestimated. The managers interviewed also indicated that NGCWs are less able to perceive hazards owing to the lack of safety understanding.

(4) Safety awareness:

Safety awareness is the underlying state of consciousness in which people notice the hazards around (Man et al., 2017), which makes people aware of which behavior foster safety (Wang et al., 2018). The majority of interviewees agreed that most unsafe behavior happens as a result of NGCWs’ own weak safety awareness. For example, B_09_ said, “…, the workers’ sense of danger prevention is relatively shallow around.” The interview data revealed that NGCWs seem to constantly blindly pursue efficiency without regard for safety and be unable to remain alert to the potential hazards during construction production activities. Choudhry and Fang (2008) pointed out that creating safety awareness within the organization is an important duty of the management team.

(5) Self-efficacy:

According to Bandura’s definition (Bandura, 1977), self-efficacy refers to NGCWs’ belief in the ability to exert control over dangerous situations in the present study. This concept is considered to be a personality trait that may greatly influence individuals’ choices of activities in different cases. NGCWs are more likely to perform dangerous tasks if they believe they can control the consequences of their actions, or hold the point that the mission is easy to complete. Some workers felt confident to perform dangerous operations because of dexterity and responsiveness; besides, they also reported that the match between their individual physical strength and the physical demands of the job made leaders inclined to assign them hazardous tasks, which further boosted their self-confidence. However, overconfidence may prompt people to set unrealistic goals and thus exhibit accident-related unsafe behavior (Salanova et al., 2012). According to the interview records, some unsafe acts happen owing to over-confidence in abilities, such as working at heights without safety harnesses and working with hands instead of tools.

(6) Safety climate:

Safety climate is a psychological perception that reflects employees’ perceived evaluation of the organization’s emphasis on safety-related issues (Fogarty and Shaw, 2010). Numerous studies have shown that employees’ safety behavior is the most frequent safety performance output of group safety climate (e.g., Lu and Tsai, 2010; Brondino et al., 2012). In this study, safety climate includes safety management commitment and workers’ safety participation. Employees’ safety behavior can be negatively affected if managers do not follow safety rules (Fogarty and Shaw, 2010). Previous interviews support this view, for example, when asked, “Don’t the managers correct you? Don’t they take safety seriously?” B_01_ answered, “If he (the manager) doesn’t wear it (safety helmet) himself, who will wear it?” Another manager interviewed expressed the viewpoint that when managers fail to prioritize safety inputs or even take the lead in not following safety rules, it will influence workers’ decision for safety behavior. It’s clear that the organization’s great attachment to safety and expectations for workers to perform tasks without compromising health may effectively promote prevention of human errors. In terms of workers’ safety participation, B_12_ said, “When others are working very carefully and cautiously, they do so themselves.” However, researchers found that NGCWs at the construction sites did not have a high level of safety participation. According to Fang et al. (2006), employees who are older, married or support more family members may have more positive perceptions of safety climate. As workers get older, their perception of safety climate may gradually increase.

(7) Safety management system:

Safety management system helps ensure effective monitoring of the company’s safety policies, procedures and practices (Gürcanli and Mungen, 2009). Most managers pointed out that without sound safety regulations, safety management would be chaotic. The response we obtained like, “Only under the constraints of regulations can the unsafe behavior of young construction workers be effectively reduced, and the safety regulation is an important factor influencing workers to make decisions about safety behavior.” In addition, safety supervision is crucial to carry out accident prevention. Respondents believe that the current site safety supervision is not well implemented. For example, when asked, “The implementation of safety supervision of workers in your project is not quite in place, is that right?” B_01_ said, “Indeed, supervision is often inadequate, so that unsafe behavior often happens and cannot get immediately stopped.” This is consistent with a finding of Man et al. (2017), the latter discovered that safety supervision is an effective way to reduce risk-taking behavior of construction workers. Moreover, to raise risk perception and understanding of the negative repercussions of unsafe behavior among NGCWs, safety training can impart vital safety knowledge, particularly information regarding the harmful consequences. Almost all of the NGCWs interviewed stated that they had gradually identified some hazardous operations after safety training.

##### Behavior Result Generation Stage

One manager noted that if nothing happened, workers would regard the choice of unsafe acts was feasible and they would continue to engage in unsafe practices. The interview transcripts and coding results validated Skinner’s reinforcement theory (Skinner, 1968). This theory, which focuses on human behavior, can plausibly explain the impact of behavior outcomes on the motivation for unsafe behavior. As a stimulus, the behavior result has a reinforcing effect on individuals. People can actively adapt to stimuli and constantly adjust their behavior according to the feedback information. Skinner divides this effect into positive reinforcement and negative reinforcement. Behavior that is positively reinforced have a greater likelihood of reappearance. Negative reinforcement means that the adverse consequences of behavior weaken or block the continuation of this behavior. In the current research, if NGCWs’ unsafe behavior does not result in a safety accident, it will increase the likelihood that the same unsafe behavior happens. On the contrary, if the consequences caused by unsafe behavior are more severe, such as property losses and serious injuries, it will weaken the possibility of the occurrence of unsafe behavior next time. During the construction of the formation mechanism model, the researchers considered the feedback of behavior result on motivation.

## Dynamic Evolution Laws About New Generation of Construction Workers’ Unsafe Behavior

### Research Method

According to Figure 1, the formation mechanism of NGCWs’ unsafe behavior is a complex dynamic system with multi-factor interactions, which requires a holistic and dynamic view. System dynamics draws on the ideas of information theory and cybernetics to improve itself, eventually becoming a discipline that studies the information feedback system. It focuses on the causal relationships between variables and observes the dynamic feedback structures of the factors using computer technologies. The applicability of this method to complicated systems has led many scholars to use it to analyze safety-related behavior of construction workers (e.g., Jiang et al., 2015; Yu et al., 2019; Ma et al., 2021) and safety management issues (e.g., Mohammadi and Tavakolan, 2019; Yu et al., 2019; You et al., 2020). Previous research has demonstrated the superiority of system dynamics in improving the understanding of intricate safety systems. Therefore, from the perspective of system evolution cycle, system dynamics was applied to reveal the dynamic evolution laws about NGCWs’ unsafe behavior, and then identify the key influencing factors, so as to lay the theoretical foundation for proposing relevant measures.

### Construction of System Dynamics Model

**FIGURE 2:**
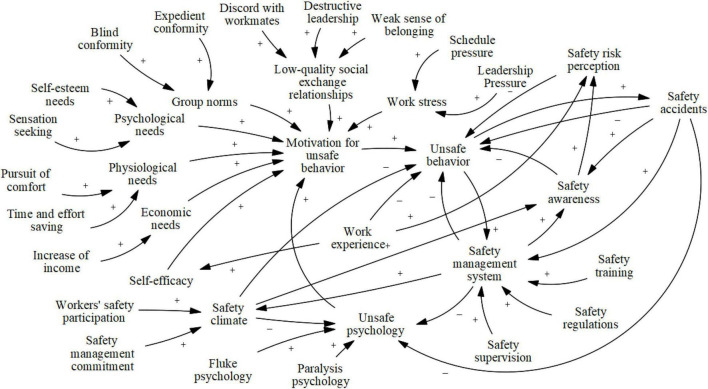
The causal diagram.

**FIGURE 3:**
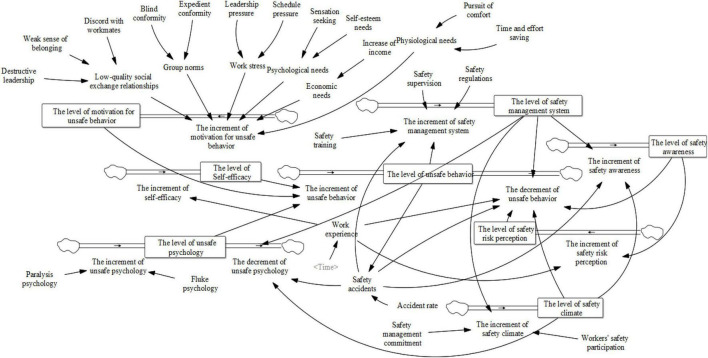
The stock flow diagram.

Before constructing the causality diagram, on the basis of the relevant literature, interview transcripts, and the formation mechanism model, the feedback relationships of the factors in the system of NGCWs’ unsafe behavior were analyzed. This process was performed through the following logic: First, motivation for unsafe behavior is the direct driver of unsafe behavior, and the link from motivation to unsafe behavior is built. Second, as shown in the formation mechanism model, internal needs and external incentives directly stimulate motivation for unsafe behavior. Thus, the links of them are built. Third, an in-depth analysis of the feedback of remaining factors, unsafe behavior and motivation for unsafe behavior is conducted to build the links of them. As a result, the causal diagram was constructed to visualize the complicated dynamic feedback of the factors, as shown in Figure 2, where “+” indicates positive feedback and “-” indicates negative feedback. In addition, on the basis of the causal diagram, the stock flow diagram (Figure 3) was established for data simulation to derive more accurate control management results. The researchers separated the categories of variables and assigned 8 state variables, 10 rate variables, 8 auxiliary variables, and 20 constants. Table 5 lists the variable names and symbols.

**TABLE 5:** Variable names and symbols.

Variable Type	Variable Name
State Variable	The level of unsafe behavior (S_1_), The level of motivation for unsafe behavior (S_2_), The level of self-efficacy (S_3_), The level of unsafe psychology (S_4_), The level of safety risk perception (S_5_), The level of safety awareness (S_6_), The level of safety climate (S_7_), The level of safety management system (S_8_)
Rate Variable	The increment of unsafe behavior (R_1_), The decrement of unsafe behavior (R_2_), The increment of motivation for unsafe behavior (R_3_), The increment of self-efficacy (R_4_), The increment of unsafe psychology (R_5_), The decrement of unsafe psychology (R_6_), The increment of safety risk perception (R_7_), The increment of safety awareness (R_8_), The increment of safety climate (R_9_), The increment of safety management system (R_10_)
Auxiliary Variable	Physiological needs (A_1_), Psychological needs (A_2_), Economic needs (A_3_), Group norms (A_4_), Low-quality social exchange relationships (A_5_), Work stress (A_6_), Work experience (A_7_), Safety accidents (A_8_)
Constant	Time and effort saving (C_1_), Pursuit of comfort (C_2_), Self-esteem needs (C_3_), Sensation seeking (C_4_), Increase of income (C_5_), Blind conformity (C_6_), Expedient conformity (C_7_), Discord with workmates (C_8_), Destructive leadership (C_9_), Weak sense of belonging (C_10_), Schedule pressure (C_11_), Leadership pressure (C_12_), Accident rate (C_13_), Fluke psychology (C_14_), Paralysis psychology (C_15_), Safety regulations (C_16_), Safety supervision (C_17_), Safety training (C_18_), Safety management commitment (C_19_), Workers’ participation in safety (C_20_).

### System Dynamics Equations and Model Parameter Settings

According to the system dynamics principles and the logical relationships among the variables, the system dynamics equation for each variable was compiled as follows:


$${\text{S}}_{1}=\text{INTEG}\left({\text{R}}_{1}-{\text{R}}_{2},\text{initial value of }{\text{S}}_{1}\right)$$

$${\text{S}}_{2}=\mathrm{INTEG}\left({\text{R}}_{3},\text{initial value of }{\text{S}}_{2}\right)$$

Where INTEG is the integral function, indicating the value of the state variable S. Other state variable equations can be given with reference to the equations of *S*_1_ and *S*_2_.


$${\text{R}}_{1}={\text{S}}_{2}\times {\text{f}}_{11}+{\text{S}}_{3}\times {\text{f}}_{12}+{\text{S}}_{4}\times {\text{f}}_{13}$$

Where *f*_11_, *f*_12_, *f*_13_ are the weights of *S*_2_, *S*_3_, *S*_4_, and *f*_11_ + *f*_12_ + *f*_13_ = 1. Other rate variable equations can be given with reference to the equation of *R*_1_.


$${\text{A}}_{1}={\text{C}}_{1}\times {\text{l}}_{11}+{\text{C}}_{2}\times {\text{l}}_{12}$$

$${\text{A}}_{7}=\text{WITH LOOK UP }\left(\mathrm{time}\right)$$

$${\text{A}}_{8}={\text{S}}_{1}\times {\text{C}}_{13}$$

Where *l*_11_ and *l*_12_ are the weights of *C*_1_ and *C*_2_. WITH LOOK UP (time) indicates the relationship between work experience and time, and the specific data were collected through the questionnaire survey. Other auxiliary variable equations can be given with reference to the equations of *A*_1_, *A*_7_, and *A*_8_.

For the case where one outcome factor corresponds to multiple cause factors, the G1 method was utilized to determine the weights of each factor, which effectively circumvents the shortcomings of hierarchical analysis process and is simple to operate without requiring consistency testing (Chu et al., 2018). Furthermore, the expert scoring method was chosen for the case where one cause factor corresponds to one outcome factor. Five corporate experts with extensive construction management experience and seven university researchers were invited to score and determine the weights of these factors. The basic information of experts is shown in Table 6, and the final weight of each factor are shown in Table 7.

**TABLE 6:** Basic information of experts (*N* = 12).

Variable	Categories	Number of Cases	Frequency (%)
Sex	Male	11	92%
	Female	1	8%
Age	Between 21 and 30	2	17%
	Between 31 and 40	3	25%
	Between 41 and 50	4	33%
	Between 51 and 60	3	25%
Degree	Bachelor	6	50%
	Master	2	17%
	Doctor	4	33%
Affiliation	Construction company	2	17%
	Consultant company	3	25%
	College and university	7	58%
Professional Title	Lecturer	2	17%
	Associate professor	3	25%
	Professor	2	17%
	Engineer	4	33%
	Senior engineer	1	8%
Work Experience	Between 6 and 10 years	2	17%
	Between 11 and 15 years	6	50%
	Between 16 and 20 years	0	0%
	More than 20 years	4	33%

**TABLE 7:** The weight of factors.

Outcome factor	Cause factor	Weight	Outcome factor	Cause factor	Weight
*R*_1_	*S*_2_	*f*_11_ = 0.375	*R*_*8*_	*A*_*8*_	*f*_81_ = 0.340
	*S*_*3*_	*f*_12_ = 0.270		*S*_*7*_	*f*_82_ = 0.355
	*S*_*4*_	*f*_13_ = 0.355		*S*_*8*_	*f*_83_ = 0.305
*R*_2_	*A*_*8*_	*f*_21_ = 0.180	*R*_*9*_	*S*_*8*_	*f*_91_ = 0.359
	*S*_*6*_	*f*_22_ = 0.210		*C*_*19*_	*f*_92_ = 0.305
	*S*_*7*_	*f*_23_ = 0.152		*C*_*20*_	*f*_93_ = 0.336
	*S*_*8*_	*f*_24_ = 0.167	*R*_*10*_	*A*_*8*_	*f*_101_ = 0.253
	*A*_*7*_	*f*_25_ = 0.143		*S*_1_	*f*_102_ = 0.130
	*S*_*5*_	*f*_26_ = 0.148		*C*_*17*_	*f*_103_ = 0.217
*R*_*3*_	*A*_1_	*f*_31_ = 0.140		*C*_*16*_	*f*_104_ = 0.207
	*A*_2_	*f*_32_ = 0.121		*C*_*18*_	*f*_105_ = 0.193
	*A*_*3*_	*f*_33_ = 0.164	*A*_1_	*C*_1_	*l*_11_ = 0.533
	*A*_*6*_	*f*_34_ = 0.204		*C*_2_	*l*_12_ = 0.467
	*A*_*5*_	*f*_35_ = 0.208	*A*_2_	*C*_*4*_	*l*_21_ = 0.459
	*A*_*4*_	*f*_36_ = 0.164		*C*_*3*_	*l*_22_ = 0.541
*R*_*4*_	*A*_*7*_	*f*_41_ = 0.833	*A*_*3*_	*C*_*5*_	*l*_31_ = 1.000
*R*_*5*_	*C*_*14*_	*f*_51_ = 0.527	*A*_*4*_	*C*_*6*_	*l*_41_ = 0.486
	*C*_*15*_	*f*_52_ = 0.473		*C*_*7*_	*l*_42_ = 0.514
*R*_*6*_	*A*_*8*_	*f*_61_ = 0.356	*A*_*5*_	*C*_*8*_	*l*_51_ = 0.282
	*S*_*7*_	*f*_62_ = 0.343		*C*_*9*_	*l*_52_ = 0.449
	*S*_*8*_	*f*_63_ = 0.302		*C*_*10*_	*l*_53_ = 0.270
*R*_*7*_	*S*_*6*_	*f*_71_ = 0.529	*A*_*6*_	*C*_*11*_	*l*_61_ = 0.480
	*A*_*7*_	*f*_72_ = 0.471		*C*_*12*_	*l*_62_ = 0.520

In order to determine the initial value of state variables and constants, the data was obtained through distributing research questionnaires to more than 200 NGCWs whose workplaces were in 13 provinces and municipalities, including Jiangsu, Henan, Shandong, Jiangxi, etc. A total of 128 valid questionnaires were returned, and the results of data analysis showed that the reliability and validity met the requirements. In addition, the data collected was dimensionless processed for the purpose of comparability. Table 8 shows the initial value of factors. Among them, the value of *C*_*13*_ was set as 0.090, which determined by Heinrich ratio (i.e., in a unit group of similar 330 accidents, 1 will result in major injury, 29 will result in minor injuries, and 300 are non-injury accidents) (Heinrich, 1931). The initial value of *A*_*7*_ was set as 0.288 through replacing work experience with work time.

**TABLE 8:** The initial value of factors.

Factor	Initial value	Factor	Initial value
*S*_1_	0.210	*C*_*8*_	0.253
*S*_2_	0.347	*C*_*9*_	0.324
*S*_*3*_	0.620	*C*_*10*_	0.290
*S*_*4*_	0.292	*C*_*11*_	0.435
*S*_*5*_	0.750	*C*_*12*_	0.390
*S*_*6*_	0.623	*C*_*13*_	0.090
*S*_*7*_	0.711	*C*_*14*_	0.290
*S*_*8*_	0.745	*C*_*15*_	0.293
*C*_1_	0.342	*C*_*16*_	0.734
*C*_2_	0.318	*C*_*17*_	0.749
*C*_*3*_	0.265	*C*_*18*_	0.751
*C*_*4*_	0.213	*C*_*19*_	0.706
*C*_*5*_	0.388	*C*_*20*_	0.715
*C*_*6*_	0.380	*A*_*7*_	0.288
*C*_*7*_	0.343		

### Simulation and Analysis

Using Vensim PLE software for simulation, and the initial level of NGCWs’ unsafe behavior was set as 0.21. Additionally, setting the parameters as follows: INITIAL TIME = 0, FINAL TIME = 12, TIME STEP = 1, SAVEPER = TIME STEP, UNITS FOR TIME = MONTH, and taking 1 year as the simulation period. Firstly, the initial state simulation of the model was conducted. Secondly, the input values of motivation for unsafe behavior, each situational factor and each individual factor were modified in turn, while keeping the input values of the remaining factors invariant to identify the key factors.

**FIGURE 4:**
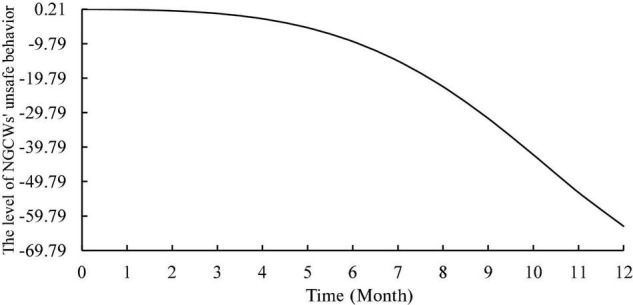
Evolution trend of NGCWs’ unsafe behavior.

In the initial state, the simulation result is indicated in Figure 4. As can be observed from Figure 4, under the synergistic effect of multiple factors, the level of unsafe behavior shows a downward trend with a three-stage characteristic, and the rate of decrease is slow first and then fast with the continuous advancement of project construction work. To be specific, in the first stage (months 0–4), the level of unsafe behavior remains the same overall with a slight decrease. The high level of unsafe behavior indicates that the safety performance of the whole system is insufficient at the beginning of the simulation. In the second stage (months 4–8), the decline rate of the level of unsafe behavior gradually accelerates. In the third stage (months 8–12), the level of unsafe behavior shows a more significant decreasing trend. It can be found that situational factors such as safety management system and safety climate have been improved over time, which has led to an improvement in NGCWs’ personal traits such as safety awareness and risk perception. The result of initial simulation validate to an opinion that a large number of construction accidents occur during the early phase of a construction project (Shin et al., 2014). In addition, although NGCWs’ unsafe behavior can be effectively curbed as time continues to pass, the apparent effect of the intervention is lagging, so the time for workers to become familiar with the intervention should be minimized.

**FIGURE 5:**
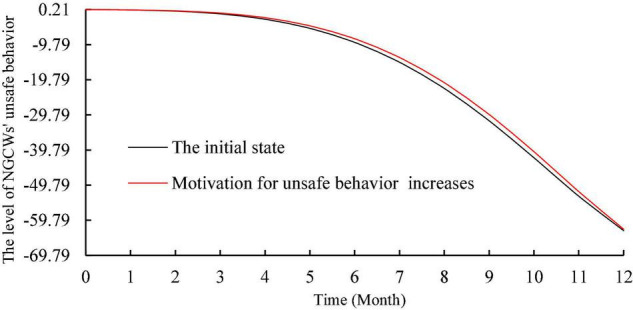
Simulation of the effect of motivation for unsafe behavior on unsafe behavior.

**FIGURE 6:**
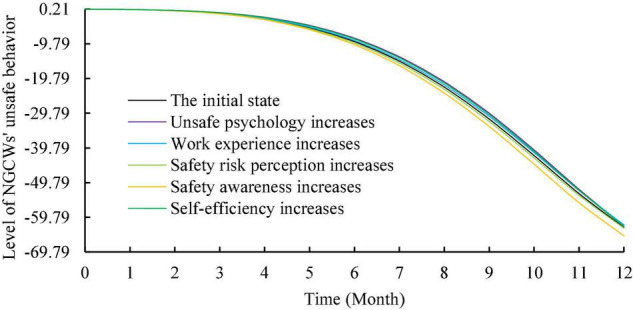
Simulation of the effect of individual factors on unsafe behavior.

**FIGURE 7:**
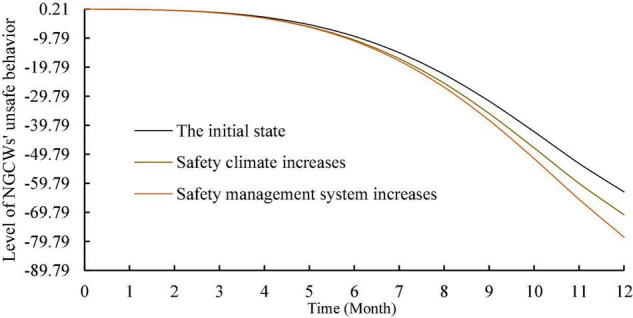
Simulation of the effect of situational factors on unsafe behavior.

To analyze the effect of motivation on the level of unsafe behavior, this study kept the input values of other factors unchanged and increased the inflow of motivation for unsafe behavior by 0.2. As shown in Figure 5, the overall trend of the level of NGCWs’ unsafe behavior increases. The input values of each individual factor and each situational factor were increased by 0.2 as well to explore their effects on the level of NGCWs’ unsafe behavior. The simulation results are shown in Figures 6, 7. In terms of individual factors, the increase of self-efficacy and unsafe psychology will lead to the increase of unsafe behavior’s level, but the effect is non-significant; the increase of safety risk perception and safety awareness will lead to the decrease of unsafe behavior’s level, and the effect of safety awareness is more significant; the effect of work experience on unsafe behavior’s level has a phased feature of decreasing in the first 2 months and increasing in the next 10 months, but the effect is relatively weak. In terms of situation factors, the increase of both safety management system and safety climate can effectively reduce unsafe behavior’s level, and the effect of safety management system is more obvious. Moreover, the simulation trend after changing the input values of each variable is similar to the initial state, which indicates that the established system dynamics model is relatively stable (Yu et al., 2019).

## Discussion

### Theoretical Contributions

First, this manuscript creatively expands the SOR model, introduces the result link and constructs the SORR behavior chain. Then, based on this theoretical framework, from the perspective of the driving force of behavior, i.e., behavior motivation, this manuscript explores the formation mechanism of NGCWs’ unsafe behavior through qualitative research. The results show that the SOR theory can effectively explain and predict unsafe behavior at construction sites. The manuscript introduces the SOR theory into the field of behavioral safety and expands the application scope of this theory. Besides, the formation mechanism of unsafe behavior mainly aims to deeply explore the logical relationship between the concerned events and the causal factors (Huang et al., 2019). This explanation of the random combination of influencing factors breaks through the limitations of the single factor analysis. In addition, previous studies on the formation process of unsafe behavior have been conducted from the perspective of cognition (Fang et al., 2016), risk perception (Huang et al., 2019), etc., and rarely from the perspective of motivation. Therefore, this study provides a new insight into how construction workers’ unsafe behavior occurs, which is a perfection and supplement to the existing literature.

Second, this study breaks through the traditional research on the static relationship between antecedent factors and unsafe behavior, analyzes the dynamic performance of construction workers’ unsafe behavior from a systematic perspective, presents the feedback structure of various factors in the system, and responds to the call for a comprehensive understanding of the potential mechanism using systems thinking (e.g., Jiang et al., 2015). The dynamic evolution laws obtained by computer simulation clearly presents the trend of change of NGCWs’ unsafe behavior that impacted by complex construction environment and multiple factors. Namely, the level of NGCWs’ unsafe behavior shows a downward trend with a three-stage characteristic and the rate of decrease is slow first and then fast during the construction period.

Third, previous research on the new generation of migrant workers has focused on city integration (Zheng, 2021), entrepreneurial willingness (Lin and Mai, 2018), entrepreneurial performance (Ma et al., 2018), etc., but has ignored the unsafe behavior of this group based on the context of China’s construction industry. The formation mechanism and dynamic evolution laws about unsafe behavior found by this manuscript enrich the body of knowledge of unsafe behavior among young construction workers. In contrast to previous studies, this study highlights the important roles played by physiological needs, psychological needs, self-efficacy, work experience and low-quality social exchange in the formation of unsafe behavior for the young construction workers in the Chinese context, which can also be clearly reflected in the laws of dynamic evolution.

### Managerial Implications

The findings also have important managerial implications for construction companies and government departments. According to the formation mechanism model, the NGCWs’ internal needs and external incentives can stimulate corresponding motivations for unsafe behavior. Hence, it is of great importance to find ways to block the emergence of motivations for unsafe behavior. In addition, based on the simulation results in the previous section, safety management system and safety climate have the most significant effect on the level of unsafe behavior. Improving the safety management system as well as creating a positive safety climate is an essential grip to curb the occurrence of NGCWs’ unsafe behavior.

Compared to older workers, younger workers usually have lower tolerance capacity. The study found that the NGCWs are likely to perform unsafe acts, such as not wearing PPE, because of the need to save time and effort and for comfort. Construction workers’ discomfort when using PPE may be caused by differences in the design and workmanship of different brands of PPE (Wong et al., 2020). One of the key takeaways for construction companies is the need to maximize safety equipment’s comfort and ease of operation while ensuring their security and reliability. This requires construction companies to increase investment in safety resources to ensure that safety facilities are adequate and reasonable. The results of the interviews suggest that self-esteem needs and sensation seeking may induce NGCWs’ hazardous acts. Therefore, effective safety education is necessary to guide them to establish the view of safety first. Besides, the economic need is one of the triggers of NGCWs’ unsafe behavior. A reasonable worker compensation system and wage increase mechanism should be set. On the basis of ensuring the basic income, workers ought to get additional compensation or job promotion opportunities according to the virtue of their work quality, professional skill level and skill qualification certificates, etc. Government departments should implement effective supervision of the payment of migrant workers’ wages and impose severe penalties on construction companies for wage defaults. Considering the unsafe influence of schedule pressure and leadership pressure, reasonable work intensity and leadership attention to safety are conducive to reducing the motivation for unsafe behavior caused by work pressure. Therefore, work tasks should be reasonably assigned and rest time should be flexibly arranged. In terms of group norms, workers should be encouraged to communicate with each other about safety and be bolstered to think independently by setting a denounce system. The results also suggest that poor quality of social exchange relationships can lead to the motivation for unsafe behavior. Construction companies need to conduct more activities to enhance the relationships of workers, co-workers, and leaders, so as to improve workers’ sense of organizational identity, belongingness and job satisfaction. Leaders also need to show concern for workers and have regular safety-related communication and exchange with them.

Furthermore, the forms of safety training can be updated to improve the effectiveness of safety training for NGCWs. Aside from the traditional centralized and indoctrination-based safety training, forms that are more likely to arouse the interest of NGCWs can be adapted, for example, VR technology can be applied to further improve the safety performance of NGCWs (Nykanen et al., 2020). Government departments also need to actively monitor the quantity and quality of safety training for construction companies and check the effectiveness of the training. In terms of safety supervision, managers at all levels should actively perform their safety supervision duties to eliminate the “formalism” of safety management. It is the responsibility of the construction company to provide sufficient human resources to maintain close safety supervision as well (Wong et al., 2020). Moreover, while construction companies continue to improve safety regulations, government departments have the responsibility to assess the safety regulations. In addition, construction companies need to pay attention to the role of safety climate in curbing NGCWs’ unsafe behavior. Safety knowledge competitions and safety meetings can be held to improve the safety climate at construction sites. A positive safety climate also influences other factors, such as safety awareness (Wang et al., 2018). Finally, interventions need to be implemented for NGCWs as early as possible to quickly and effectively curb their unsafe acts.

### Limitations and Future Work

The manuscript still has some shortcomings and deserves further improvement in future studies. First, grounded theory still has the risk of confirmation bias to some extent. In future studies, the researchers’ interviewing and coding skills could be further enhanced to identify some information that might be missed. Second, comparative analysis between the new and old generations of construction workers is meaningful, and either qualitative or quantitative research methods can be used to conduct controlled analyses to identify specific differences in unsafe behavior between the two groups and to propose more targeted improvement measures. Third, the data collected from the questionnaire survey and expert scoring method are somewhat subjective in assigning values to some of the variables in the system dynamics model, and future research can adopt a more objective approach to improve the accuracy of the simulation results.

## Conclusion

At present, the research on the formation mechanism of construction workers’ unsafe behavior from the perspective of behavior motivation is still in the early stage. As a major component at construction sites and an important driver of China’s current economic development, the NGCWs should receive more attention about their safety. In this regard, this manuscript portrays the formation mechanism of their unsafe behavior based on grounded theory. In this process, the SOR theory is expanded to provide a suitable research framework. In addition, based on systems thinking, system dynamics models of NGCWs’ unsafe behavior are constructed to explore the dynamic evolution laws and the effect of influencing factors. The conclusions can be drawn as follows:

(i) The formation process of NGCWs’ unsafe behavior involves three stages, including behavior motivation stimulation stage, behavior motivation externalization stage, and behavior result generation stage. Motivations for unsafe behavior can be stimulated by internal needs (i.e., economic, physiological, and psychological needs) and external incentives (i.e., work stress, group norms and low-quality social exchange relationships). Influencing factors in the decision-making process of externalizing motivation into behavior include individual factors (i.e., work experience, self-efficacy, safety risk perception, unsafe psychology, and safety awareness), situational factors (i.e., safety management system and safety climate) and behavior result.
(ii) Under the synergy of various factors, with the continuous progress of project construction, the level of NGCWs’ unsafe behavior tends to decrease, and the decline rate is slow first and then fast. The increase of the motivation for unsafe behavior will aggravate the occurrence of unsafe behavior. Improving both individual factors and situational factors can reduce the level of NGCWs’ unsafe behavior, and the role of situational factors is more obvious.

## Data Availability Statement

The raw data supporting the conclusions of this article will be made available by the authors, without undue reservation.

## Author Contributions

GN contributed to conception and design of the study. LL, SW, XM, and QL collected the data. LL and SW contributed to the data analysis. LL, SW, and XM contributed to the original draft of the manuscript. GN, LL, YF, and QL contributed to the review and editing of the manuscript. All authors have read and agreed to the Published version of the manuscript.

## Conflict of Interest

The authors declare that the research was conducted in the absence of any commercial or financial relationships that could be construed as a potential conflict of interest.

## Publisher’s Note

All claims expressed in this article are solely those of the authors and do not necessarily represent those of their affiliated organizations, or those of the publisher, the editors and the reviewers. Any product that may be evaluated in this article, or claim that may be made by its manufacturer, is not guaranteed or endorsed by the publisher.

## Appendices

**Appendix A.** Outline of the interview with the new generation of construction workers (NGCWs).

Q1. Have you ever experienced or heard of some safety accidents that impressed you during your work? Can you explain the incident in detail?
Q2. Do you think these safety accidents are caused by human factors or objective factors?
Q3. Can you explain what is unsafe behavior? What unsafe behavior do you have in your daily work?
Q4. Why do you perform these actions since you know they are unsafe? Please list at least three reasons.
Q5. Would you do this in any situation? When would you not perform these unsafe acts?
Q6. What unsafe behavior exists among the young workers around you? Why do they do this?
Q7. Have you ever considered the consequences of unsafe behavior? What do you think the impact of unsafe behavior will be on yourself, workmates and family?
Q8. Assuming that these unsafe behaviors resulted in injury or death to others or yourself, how would your intentions and attitudes toward similar behaviors be affected?

**Appendix B.** Outline of the interview with the grassroots manager.

Q1. What is the proportion of NGCWs at the construction site you are responsible for? What is the difference between them and the old generation of construction workers?
Q2. How do you think NGCWs performs at work? What are the characteristics of their work attitude and work behavior?
Q3. What unsafe behavior occurs among NGCWs at project sites and how frequently it occurs?
Q4. Why would they perform unsafe behavior? What are the possible motivations and reasons? You can give examples.
Q5. What is the impact of their unsafe behavior?
Q6. What measures does your project department have to deal with the NGCWs’ unsafe behavior?
Q7. What else can be done to reduce NGCWs’ unsafe behavior?
